# Transcultural validation of the return-to-work self-efficacy scale in Korean patients with work-related injuries

**DOI:** 10.1186/s12889-020-08979-w

**Published:** 2020-06-03

**Authors:** Jeong-Eun Lee, Su Bin Yoo, Ja-Ho Leigh

**Affiliations:** 1 Biomedical Research Institute, Seoul National University Hospital, Seoul, Republic of Korea; 2 Coresearch Coop, Seoul, Republic of Korea; 3 Department of Rehabilitation Medicine, Seoul National University Hospital, Seoul National University College of Medicine, 101, Daehak-ro, Jongno-gu, Seoul, 03080 Republic of Korea; 4 Department of Rehabilitation Medicine, Korea Workers’ Compensation and Welfare Service Incheon Hospital, Incheon, Republic of Korea

**Keywords:** Return to work, Self-efficacy, Work-related injuries, Rehabilitation, Factor analysis

## Abstract

**Background:**

This study aimed to develop a Korean version of the Return-to-Work Self-Efficacy (RTWSE)-19 Scale using forward- and backward-translation and investigate the validity of the RTWSE Scale specifically for Korean workers with work-related injuries.

**Methods:**

Participants were 202 injured workers who had filed a claim accepted by the workers’ compensation system and had received medical rehabilitation at workers’ compensation hospitals following a work-related musculoskeletal injury. Among these participants, 88.1% were male, 54.5% were over 45 years, 45.5% were manufacturing employees, and 54.5% were craft or machine operator and assemblers. The 19 item RTWSE-19 scale was developed by Shaw et al. and have three underlying subscales: (i) meeting job demands, (ii) modifying job tasks, and (iii) communicating needs to others. Statistical analysis included exploratory factor analysis (maximum likelihood estimation with oblique quartimin rotation), internal consistency reliability using Cronbach’s alpha, and correlations with related measures: pain intensity; fear-avoidance beliefs; general health; depression; and general self-efficacy.

**Results:**

Using exploratory factor analysis, three factors with 17 items were identified: meeting job demands, modifying job tasks, and communicating needs to others. The removal of two items in the modifying job tasks domain resulted in an increased reliability. The Korean version of the RTWSE-17 showed reasonable model fit (CFI = .963; TLI = .943; RMSEA = .068; SRMR = 0.029), satisfactory reliability (*r* = 0.925), no floor and ceiling effect, and construct validity.

**Conclusions:**

The Korean RTWSE-17 scale was found to possess good psychometric properties and could address different injury types ranging from fractures to amputations involved in sub-acute and rehabilitation phases in the Korean context. This study’s findings provide insights for practitioners and researchers to return to work after rehabilitation in a Korean clinical and workplace setting.

## Background

The return-to-work (RTW) process after experiencing a work-related injury is complex and affected by various factors other than physical ability [1–3]. In a systematic literature review, approximately 100 different determinants were identified as influencing factors on RTW outcomes [4]. While injury- or disability-related factors, such as pain and functional status, are important determinants of successful work reintegration, evidence increasingly suggests that psychosocial factors are strong predictors of RTW outcomes. A major psychosocial factor in the RTW process is a worker’s expectation of recovery, or return-to-work self-efficacy (RTWSE) [2, 5, 6]. Higher levels of RTWSE have consistently been associated with better RTW outcomes for workers with all-cause sickness absences [7, 8].

RTWSE is based on the Readiness for Return to Work Model, proposed by Franche and Krause,[6] which combine the Phase Model of Occupational Disability and the Readiness for Change Model. The former model addresses the influence of physical and psychological factors in specific phases of disability: acute, sub-acute, and chronic. The latter model concerns the motivational factors that instigate and maintain behavioral changes. In the Readiness for Return to Work Model, which regards RTW as a health-related behavior, RTWSE is defined as “the personal judgment of one’s ability to do whatever is necessary to return to work” [9]. This concept is useful in understanding the motivational and pain-management aspects of RTW. Shaw and Huang [9] emphasize clinical intervention focused on self-efficacy for functional recovery and pain control are needed for an early RTW.

In initial studies on RTW, measurements of general self-efficacy or self-efficacy for managing pain were mainly used, and there were conflicting findings on the impact of self-efficacy in RTW, as no self-efficacy measurement specific to RTW existed [10]. Since 2010, RTWSE scales specific to resuming work after the onset of a work-related injury have been developed and validated, especially for injured workers with musculoskeletal injuries [10–14], mental illness [15, 16], and psychological injuries [17, 18].

RTWSE-19, based on qualitative research findings by Shaw and colleagues [9, 14], is a self-report questionnaire assessing workers’ confidence in their RTW abilities. This validated scale consists of 19 items and three subscales: meeting job demands; modifying job tasks; and communicating needs to others. While self-efficacy is generally conceptualized as an individual-level factor, this scale reflects both the personal motivation and workplace barriers of workers [14]. Shaw and colleagues [14] suggested that the RTWSE-19 could be useful in the evaluation of the effectiveness of clinical and workplace interventions and in exploring mediating RTW mechanisms. Most empirical studies on RTWSE scale development have focused on the acute disability phase and low back pain (LBP) [11, 12, 14]. Studies have reported that the RTWSE-19 scale had acceptable validity and reliability [11] and that RTWSE was directly related to RTW outcomes in workers suffering from LBP [5].

This study aimed to develop a Korean- version of the RTWSE-19 through a forward- and backward-translation process and to examine the scale’s psychometric properties using a Korean sample of workers with occupational injuries. Specifically, we evaluated the scale’s factor structure, internal consistency, floor and ceiling effects, and the construct validity.

## Methods

### Participants and procedures

Participants were injured workers who had filed workers’ compensation claims that had been accepted following work-related musculoskeletal injuries in Korea. Claimants were eligible if they were: (i) absent from work due to a musculoskeletal injury sustained at work ranging from a fracture to an amputation; (ii) in treatment, such as a sub-acute intensive rehabilitation program, tailored-exercise program, or work hardening program at Korea Worker’s Compensation and Welfare Service (KCOMWEL) hospitals; (iii) aged 18–65 at the time of the survey; (iv) without central nervous complications; (v) able to understand and speak Korean.

A face to face survey was performed from September 2016 to September 2017. Rehabilitation physicians and physical therapists at six KCOMWEL hospitals were approached to assist with the recruitment of eligible participants. These physicians explained the purpose and process of the survey with information sheets about the study to the eligible patients and listed those who verbally consented to participate. The interviewers visited the centers and conducted face-to-face surveys within 2 weeks after recruitment took place. The interview was conducted by the authors of this study along with trained social work graduate students. At the time of the interview, the participants were reassessed for eligibility, the details of the study were re-explained, and they were asked to provide consented to participate again. They were then asked questions regarding demographic information, RTWSE, and other variables. Participants were compensated with about 45 US dollars for completing the survey. Ultimately, 254 injured workers participated, of which 202 were under treatment (79.5%) and 52 had returned to work after treatment (20.5%).

### Translation procedure

A forward- and backward-translation procedure was applied to translate the RTWSE-19 from English to Korean. After receiving written permission from the corresponding author of the RTWSE-19 scale [14], a forward translation was made of the original English version of the scale into Korean. Two Korean versions were made: one by a professional bilingual translator, and another by a bilingual rehabilitative medicine doctor. The two Korean translations were then unified by the authors after discussing conceptual equivalence and cultural differences. The Korean scale was reviewed by a Korean language and literature PhD candidate, and it was then translated back into English by a different professional translator who was unfamiliar with the original English version. The discrepancies between the back-translated scale and the original English-language scale were reviewed and the backward-translation version was discussed, while minor errors in items 5 and 17 were highlighted. In the original item 5, the word “job performance” (“Could meet expectations for job performance”) was back-translated into “job duties.” The term “job performance” was intended to be formal written evaluation of employees’ work, and the Korean word for “job performance” was corrected after discussion. The original item 17 (“Could discuss openly with your supervisor things that may contribute to your discomfort”) lacked the word “supervisor”; it was corrected.

### Measures

*RTWSE* was measured using the RTWSE-19, which is intended to assess workers’ self-reported confidence about resuming work after the onset of LBP. The scale consists of 19 items under three subscales: (i) meeting job demands (7 items); (ii) modifying job tasks (7 items); (iii) and communicating needs to others (5 items). The response range is from 1 (not at all confident) to 10 (totally confident). The scale was validated through exploratory factor analysis and had high internal consistency for the overall scale and the three sub-scales. Average scores of the items were computed; higher scores indicated more confidence about returning to work.

*Pain intensity* was assessed using an item from the intensity subscale of the Von Korff pain scale [19]. Participants were asked to rate their pain intensity on an 11-point numeric rating scale from 0 (“no pain at all”) to 10 (“worst pain possible”) for currently experienced pain and the average amount of pain experienced during the past month respectively.

*Fear-avoidance beliefs* were assessed using the Fear-Avoidance Beliefs Questionnaire (FABQ) [20], which is an 11-item scale designed to measure a person’s beliefs about how physical activity and work influence their back pain. The scale consists of two subscales: physical activity (FABQ-P); and work (FAB-W). Each item was answered on a 7-point Likert scale ranging from “strongly disagree” to “strongly agree.” This scale was adapted to evaluate health-related beliefs in patients and workers with upper limb and neck pain in several studies [21, 22].

*General health* was assessed using the Short Form-12 (SF-12), which is a 12-item version of the Short Form-36 [23] that measures physical and mental health. Scores range from 0 to 100, where higher scores indicate better health. Scores were computed using QualityMetric Health Outcomes scoring software 4.0.

*Depression* was measured with the Patient Health Questionnaire (PHQ-9) [24] which assesses depressive symptoms and consists of 9 items on a 4-point Likert scale.

*General self-efficacy* was assessed using the General Self-Efficacy Scale (GSE) [25]. The GSE is 10-item scale measuring an individual’s sense of personal competence to deal effectively with a variety of stressful situations. Each item was rated from 1 (“not at all true”) to 4 (“exactly true”), yielding total scores between 10 and 40.

### Statistical analysis

An exploratory factor analysis (EFA) was conducted to examine the factor structure of the RTWSE-19 scale for the work-related injured worker sample in Korea. EFA was applied because the RTWSE-19 scale had not previously been developed and administered to a Korean sample and such an analysis was necessary to explore the subscale structure. There are several opinions and guiding rules of thumb for the sample size for factor analysis [26]; of at least 300 cases [27], 100 or greater [28], where 100 is considered poor, 200 as fair, 300 as good, 500 as very good, and 1000 or more as excellent [29]. Some recommendations suggest the sample to variable ratio as a rule of thumb range from 5:1 [27], 10:1 [30], and 20:1 [28]. The sample size in this study is considered proper on the basis of some guidelines, because it is made up of 202 participants, the sample to variable ration is about 10:1. To determine the number of factors, factor structures were examined for factors numbered 1, 2, 3 and 4, and the appropriate factor structure was initially determined by model fit indices and simple loading patterns. If the largest factor loading was below 0.4, and if items were loaded above 0.4 with more than one factor (cross-loaded), items were removed. Goodness-of-fit measures, including the comparative fit index (CFI), Tucker-Lewis index (TLI), root mean square error of approximation (RMSEA), and standardized root mean square residual (SRMR) were used to assess fit. The minimal requirements for adequate model fit were: CFI and TLI values greater than .90 (> 0.90: reasonable model fit, > 0.95: good model fit), and RMSEA and SRMR values less than .08 (< 0.08: reasonable model fit, < 0.05: good model fit) [31]. EFA was conducted using maximum likelihood estimation and oblique quartimin rotation to allow for correlation between factors. Missing data were not present in the sample.

Additionally, possible floor and ceiling effects were identified. A floor or ceiling effect is usually defined as more than 15% of respondents achieving the lowest or highest score level respectively [32]. The presence of floor or ceiling effects indicates that extreme items are missing in the lower or upper end of the scale, indicating limited content validity. Reliability was analyzed using Cronbach’s alpha to measure internal consistency. Pearson’s correlation coefficient was used to evaluate the construct validity of the overall RTWSE-19 scale and its subscales with several validating measures: pain intensity; fear-avoidance beliefs; general health; depression; and general self-efficacy. Correlation coefficients were evaluated with respect to guidelines presented by Drummond, Sheperis, and Jones, indicating that a correlation greater than .50 is considered a very high correlation, 0.4 to 0.49 is a high correlation, .21 to .40 is moderate or acceptable, and less than .20 is low and unacceptable [33]. Strong correlations between the RTWSE-19 and related construct (i.e. r > 0.5) were considered evidence of convergent construct validity.

MPlus Version 5.21 (Muthén & Muthén, Los Angeles, CA) was used for the EFA, and IBM SPSS Statistics 21.0 was used for the descriptive statistics.

## Results

### Sample characteristics

Among the participants, 88.1% were male, 54.5% were over 45 years old, 84.2% had more than a high-school education, 45.5% worked in manufacturing, 54.5% were craft or machine operator and assemblers, 65.3% were regular workers, and 70.3% worked in small- or medium-sized organizations. Before the interview, 31.7% had been absent from work for around 4–6 months and 23.8% had been for 7–9 months, with total participants at an average of 7 months of sickness absence. Fractures represented the largest portion (61.4%) of primary injuries, followed by cartilage or tendon rupture (23.8%). More than half of the participants had injured their lower extremities (47.5%), spine (45.0%), or upper extremities (30.7%) (Table 1).

**Table 1:** Demographic and work-related characteristics of study participants

Variable	Participants of this study		Korean Workers’ Compensation statistics (%)^a^
	N	%	
Gender			
Male	178	88.1	79.7
Female	24	11.9	20.3
Age (years)			
≤ 39	47	23.3	28.8
40–49	66	32.7	24.1
50–59	72	35.6	33.1
≥ 60	17	8.4	14.1
Education			
< High school	32	15.8	22.5
High school	97	48.0	50.6
≥ College/university	73	36.1	26.9
Occupational categories			
Manufacturing	92	45.5	37.9
Wholesale/retail/accommodation/food	34	16.8	10.8
Construction	53	26.2	20.7
Others	23	11.5	30.6
Job categories			
Manager/professionals/clerks	34	16.9	14.2
Craft workers/Plant, machine operators/assemblers	110	54.5	55.4
Elementary workers	44	21.8	18.7
Others	14	6.9	11.7
Employment type			
Regular worker	132	65.3	70.3
Temporary worker	25	12.4	10.1
Day worker	43	21.3	19.4
Self-employed/employer	2	1.0	0.2
Organization size			
Small	49	24.3	38.7
Medium	93	46.0	52.8
Large	57	28.2	8.5
Don’t know	3	1.5	
Duration of sickness absence			
≤ 3 months	22	10.9	36.8
3-6 months	64	31.7	33.7
7–9 months	48	23.8	18.8
10–12 months	17	8.4	3.9
≥ 13 months	51	25.2	6.9
Hospital service use type			
Inpatient	59	29.2	
Outpatient	143	70.8	
Types of main injury			
Sprain and strain	13	6.4	
Peripheral nerve injury only	5	2.5	
Rupture of cartilage or tendon	48	23.8	
Fracture	124	61.4	
Amputation	10	5.0	
N/A	2	1.0	
Injured area of the body (more than one)			
Spine	91	45.0	
Upper extremities	62	30.7	
Lower extremities	96	47.5	

Note: ^a^Those who had been evaluated for return to work with first wave of data from the second cohort of panel study of Korean Worker’s Compensation Insurance (PSWCI) in 2018

The characteristics were comparable to the descriptive statistics with first wave of data from second cohort of panel study of worker’s compensation insurance (PSWCI) organized by KCOMWEL. PSWCI is an open data source with a representative sample of 3294 participants. The population of 75,392 injured workers who had completed medical care and benefit in 2017 stratified by gender, age, region, grade of disability and recovery service used. We selected those who had been evaluated for RTW, because they aligned closest to our inclusion criteria of participants, and we compared this with our study’s sample. When comparing the two samples, those who had been evaluated to RTW among all workers with work-related injuries, the proportion of men, college graduates, large enterprise workers, manufacturing and construction workers, and those who had been absent from work for more than 1 year was higher in the sample of this study. This difference is due to relatively more patients with severe injury in long-term medical care in rehabilitation centers of KCOMWEL hospitals, which are more likely to be males working in manufacturing or construction industry.

### Descriptive statistics of the RTWSE-19

As shown in Table 2, the mean scores of each item of the RTWSE-19 ranged from 4.72 ± 3.38 to 8.09 ± 2.39. The two items with the highest mean scores were item 16 (“Could you describe to your supervisor the nature of your injury and your medical treatment”) and item 17 (“Could you discuss openly with your supervisor things that may contribute to your discomfort”) with mean scores of 8.09 ± 2.39 and 7.97 ± 2.48 respectively. The two items with the lowest mean scores were item 3 (“Could you change the type of work activities you do to reduce discomfort”) and item 14 (“Could you reduce your physical workload”) with mean scores of 4.72 ± 3.38 and 4.81 ± 3.13 respectively. Items related to the “communicating needs to others” subscale showed relatively higher mean scores, while items related to the “modifying job tasks” subscale featured relatively lower mean scores.

**Table 2:** Items summary data and final solution of an exploratory factor analysis for the RTWSE scale

RTWSE scale Item		Mean	SD	Factor loadings (S.E) ^a^		
				Factor1	Factor2	Factor3
**Meeting Job Demands**						
2	Fulfill all of your duties and responsibilities?	6.28	3.09	0.65 (0.07)	0.08 (0.08)	− 0.01 (0.05)
5	Meet expectations for job performance?	5.96	2.78	0.50 (0.07)	0.22 (0.08)	0.12 (0.07)
6	Perform most of your daily activities at work?	5.55	2.97	0.70 (0.06)	−0.04 (0.08)	0.01 (0.05)
9	Keep up with the pace at work?	5.37	2.80	0.94 (0.04)	− 0.08 (0.08)	− 0.08 (0.07)
13	Meet your production requirements?	5.52	3.11	0.86 (0.04)	0.07 (0.07)	0.02 (0.04)
15	Do everything you’re trained to do?	5.79	3.16	0.82 (0.04)	0.10 (0.07)	0.03 (0.05)
18	Do your work without slowing others down?	5.59	2.91	0.93 (0.03)	−0.00 (0.03)	−0.06 (0.06)
**Modifying Job Tasks**						
1	Suggest to your supervisor ways to change your work to reduce discomfort?	5.50	3.14			
3	Change the type of work activities you do to reduce discomfort?	4.72	3.38	−0.06 (0.11)	0.72 (0.07)	−0.04 (0.08)
7	Avoid re-injury?	6.83	3.04			
10	Modify the way you work to reduce discomfort?	5.88	3.17	0.22 (0.10)	0.69 (0.07)	−0.02 (0.04)
12	Avoid activities that are likely to increase pain?	5.85	3.15	0.10 (0.10)	0.51 (0.08)	0.27 (0.07)
14	Reduce your physical workload?	4.81	3.13	0.25 (0.09)	0.58 (0.07)	0.02 (0.05)
19	Request changes in your workstation or work area to reduce discomfort?	5.68	3.28	−0.01 (0.02)	0.67 (0.05)	0.24 (0.07)
**Communicating Needs to Others**						
4	Explain any physical limitations you may have to your co-workers?	7.77	2.67	−0.08 (0.08)	0.02 (0.07)	0.64 (0.06)
8	Get co-workers to help you with activities that might cause discomfort?	7.26	2.84	0.19 (0.08)	0.30 (0.08)	0.41 (0.07)
11	Get emotional support from coworkers (such as listening or talking about your problem)?	7.31	2.66	0.07 (0.08)	0.15 (0.08)	0.53 (0.07)
16	Describe to your supervisor the nature of your injury and your medical treatment?	8.09	2.39	−0.12 (0.07)	−0.01 (0.02)	0.89 (0.04)
17	Discuss openly with your supervisor things that may contribute to your discomfort?	7.97	2.48	0.00 (0.01)	−0.06 (0.07)	0.95 (0.04)

### Evaluation of psychometric properties

#### Exploratory factor analysis

EFA models of 1–4 factors revealed that a 3-factor model was the best fit for the 19-item scale (Χ^***2***^ = 228.834, *p* < .000; CFI = .953; TLI = .931; RMSEA = .069; SRMR = 0.032), but low factor loading (below 0.4) was indicated for item 1 (“Could you suggest to your supervisor ways to change your work to reduce discomfort?”) and item 7 (“Could you avoid re-injury?”).

After removing two items from the item pool, EFA was conducted for 1–4 factor models. The 3-factor model of the 17-item scale demonstrated reasonable model fit, with marginal improvement of fit-index values (Χ^***2***^ = 169.401, *p* < .000; CFI = .963; TLI = .943; RMSEA = .068; SRMR = 0.029) compared to 3-factor model of the 19-item scale. The resulting screen test also suggested a 3-factor solution. The final model revealed 3 distinct concepts: meeting job demands (7 items); modifying job tasks (5 items); and communicating needs to others (5 items). Two items from the original 19-item scale which concerned modifying job tasks were excluded from the final model. The factor loadings for each item are presented in Table 2.

#### Intercorrelations of subscales

The subscales were significantly and moderately correlated: meeting job demands and modifying job tasks (*r* = 0.612, *p* < .001); meeting job demands and communicating needs (*r* = 0.494, *p* < .001); and modifying job tasks and communicating needs (*r* = 0.501, *p* < .001) (Table 3).

**Table 3:** Descriptive statistics, floor and ceiling effects and reliability of the Korean version of RTWSE

Scale	Mean	SD	N (%) at floor	N (%) at ceiling	Cronbach’s alpha	Intercorrelations		
						(1)	(2)	(3)
Total RTWSE-17 score	6.20	1.99	0 (0.0)	3 (1.5)	0.925	.893 (<.001)	.842 (<.001)	.743 (<.001)
(1) Meeting job demand	5.72	2.48	5 (2.5)	8 (4.0)	0.926	1	.612 (<.001)	.494 (<.001)
(2) Modifying job task	5.39	2.55	10 (5.0)	7 (3.5)	0.851		1	.501 (<.001)
(3) Communicating needs	7.68	2.05	0 (0.0)	25 (12.4)	0.842			1

#### Floor and ceiling effect

No floor or ceiling effects were found for total RTWSE and subscale scores using the criteria of 15%. Regarding communicating needs RTWSE, 12.4% achieved the highest score (10), below the 15% cutoff (Table 3).

#### Reliability

Cronbach’s alpha for all of the overall scales and subscales was satisfactory. The Cronbach’s alpha for the overall RTWSE-17 was 0.925 and was 0.842 for communicating needs, 0.851 for modifying job tasks, and 0.926 for meeting job demands (Table 3).

### Construct validity

Significant correlations were found between fear-avoidance beliefs about physical activity (*r* = − 0.231, *p* < 0.001) and work (*r* = − 0.441, *p* < 0.001), SF-12 mental health (*r* = 0.324, *p* < 0.001), depression (*r* = − 0.301, *p* < 0.001), and general self-efficacy (*r* = 0.502, *p* < 0.001) and RTWSE-17 scores. Current pain intensity (*r* = − 0.028, *p* = .692) and SF-12 physical health (*r* = 0.061, *p* = .386) showed no correlation, and the monthly average pain intensity (*r* = − 0.150, *p* = 0.033) showed low correlation with RTWSE-17 scores. These patterns did not differ in significance or direction when applied to the subscales of the RTWSE-17, except that physical fear-avoidance showed no correlation with modifying job tasks and communicating needs (Table 4).

**Table 4:** Correlations between the Korean version of RTWSE and relevant constructs

	Total RTWSE-17 score		Meeting job demands		Modifying job task		Communicating needs	
Current Pain	−.028	(.692)	−.062	(.380)	−.031	(.664)	.052	(.463)
Average Pain	−.150	(.033)	−.196	(.005)	−.107	(.128)	−.026	(.713)
Fear avoidance, physical activity	−.231	(<.001)	−.301	(<.001)	−.116	(.101)	−.106	(.135)
Fear avoidance, work	−.441	(<.001)	−.489	(<.001)	−.300	(<.001)	−.252	(<.001)
SF12, physical Health	.061	(.386)	.117	(.096)	.060	(.400)	−.074	(.294)
SF12, mental Health	.324	(<.001)	.335	(<.001)	.179	(.011)	.282	(<.001)
Depression	−.301	(<.001)	−.298	(<.001)	−.140	(.047)	−.311	(<.001)
General self-efficacy	.502	(<.001)	.479	(<.001)	.285	(<.001)	.495	(<.001)

## Discussion

To validate the RTWSE-19 for individuals with work-related injuries in Korea, the scale was forward and backward translated and its psychometric properties were evaluated. The Korean version of the RTWSE-19 scale revealed good psychometric properties, reliability, and construct validity.

The three factors with 17 items were identified: (i) meeting job demands, (ii) modifying job tasks, and (iii) communicating needs to others. All of the items (except two which were removed) were classified into the same constructs as in the original RTWSE-19 [13]. The two excluded items (suggesting changes to supervisor and avoiding re-injury) were removed due to low factor loading, though both were originally included in the modifying job tasks subscale. These results may reflect workers’ relative lack of decision-making authority or control over their work in the Korean corporate environment. Providing work accommodations or taking steps to prevent accidents and returning to pre-injury jobs seems to be beyond the control of workers and entirely subject to the employers’ will and discretion. Furthermore, injured workers were reluctant to present needs for work accommodations to return to pre-injury jobs to employers or supervisors because it might lead to penalties or problems in the workplace, and workers recognized their lack of control over many aspects of the RTW process [9, 34, 35].

All RTWSE total and subscale scores remained below the 15% threshold for floor and ceiling effects, which indicated adequate content validity. Of respondents, 12.4% achieved the highest score in the communicating needs subscale, but no ceiling effect was identified, unlike the Danish version of the RTWSE-19 [12].

The RTWSE-17 demonstrated good internal consistency reliability with a Cronbach’s alpha coefficient of 0.925, similar to the original scale’s 0.955 [14]. The meeting job demands subscale had the highest Cronbach’s alpha coefficient at 0.926 and the communicating needs subscale had the lowest at 0.842. A similar pattern was reported in the Danish version, with meeting job demands having the highest Cronbach’s alpha at 0.97 and communicating needs having the lowest at 0.93.

Finally, the strongest correlation was found between general self-efficacy and the total RTWSE-17 score in a construct validity test. The general sense of self-efficacy, core self-evaluation, is a relevant construct concerning RTWSE in broad contexts [15, 36] and is a significant factor in returning to work [7, 37–39]. The similar correlation strengths between RTWSE and general self-efficacy found in the RTWSE validity study [15] also supports our result.

Total RTWSE-17 scores showed very weak correlation with pain intensity and SF-12 physical health; in particular, current pain intensity and SF-12 physical heath failed to display statistical significance. The lack of correlation corresponds with the findings of the original study [14], which showed little or no relationship between pain constructs and functional limitations; this contrasts with the findings of Brouwer and colleagues [10]. They found that high levels of current pain and more impaired levels of SF-12 physical health were associated with lower pain RTWSE scores. Pain RTWSE refers to the belief in one’s ability to manage persistent pain. The conflicting findings suggest two related explanations regarding our study results: perceived levels of pain intensity and functional limitations themselves did not directly impact workers’ beliefs concerning their current ability to perform their job responsibilities, but that these beliefs were mediated by pain management and coping abilities; and the two items removed from modifying job tasks subscale were more likely to be associated with the construct of pain RTWSE. The latter explanation can be supported by a biopsychosocial RTW model, where pain does not directly predict disability outcomes, but psychosocial factors mediate individuals’ reactions to injuries [5]. Our study found inconstant correlation between perceived current and average (during the past month) pain intensity and RTWSE. Although the measurement of subjective pain intensity was confirmed to yield similar results [40], it can be understood that asking about the average pain intensity experienced during the past month produces meaningful results for severely injured patients with chronic pain on long-term sick leave, unlike for workers with acute LBP as in previous studies [10, 14]. Further analysis of the relationship between pain and RTWSE that accounts for measurement and population issues is needed.

### Strengths and limitations

In this study, the participants were mostly patients with musculoskeletal injuries ranging from fractures to amputations with sub-acute and rehabilitation phase, while the original scale was developed using a sample of patients experiencing acute, work-related LBP. This study confirmed that the RTWSE scale is applicable to the expanded sample, in different socioeconomic and cultural environments, and with a broad spectrum of work injuries, severity levels, and disability phases. Black and colleagues [17] examined the RTWSE scale for both upper-body musculoskeletal and psychological work-related injuries and found no difference between these types of injury in the work completion beliefs subscale, which is related to meeting job demands.

This study has some limitations. First, the sample in this study had a wide variety of injuries and severity ranged from sprains and strains to amputations, and they experienced relatively long-term sickness absences (median = 7 months, range = 1–55 months). The result of additional analysis showed U-shape pattern of RTWSE score, in that those who were absent for less than 3 months and more than 13 months were more likely to have higher RTWSE than the other group. However, cases of some categories were so small that there is a limit to interpretation. Differences in RTWSE according to these characteristics (injury type, severity, and duration of work disability) must be examined in further studies. Second, a retest was not conducted to determine the scale’s responsiveness in detecting changes over time as shown by test-retest reliability. Third, no follow-up was conducted after the initial investigation, so this study did not address the scale’s validity regarding the RTWSE’s predictiveness of actual RTW. Due to the fact that actual RTW is an important outcome measure, follow-up investigations and a predictive validity analysis of RTWSE should be conducted in future studies to understand the underlying mechanisms for RTW and implications for RTW interventions.

## Conclusion

The Korean version of RTWSE, consisting of 3 subscales and 17 items, resulted from a Korean translation of the RTWSE-19. The RTWSE-17 showed acceptable psychometric properties, satisfactory reliability, and construct validity in a sample of Korean workers with work-related musculoskeletal injuries ranging from fractures to amputations in sub-acute and rehabilitation phases. This study suggested that the instrument can address different injury types and different disability phases in clinical settings. Future studies should confirm the validity of the scale and evaluate the effectiveness of RTW interventions.

## Data Availability

The datasets generated during and/or analyzed during the current study are available from the corresponding author on reasonable request.

## Abbreviations

RTW: Return-to-work
RTWSE: Return-to-work self-efficacy
LBP: Low-back pain
KCOMWEL: Korea Worker’s Compensation & Welfare Service
FABQ: Fear-Avoidance Beliefs Questionnaire
SF-12: Short Form-12
PHQ-9: Patient Health Questionnaire
GSE: General Self-efficacy Scale
EFA: Exploratory factor analysis
CFI: Comparative fit index
TLI: Tucker-Lewis index
RMSEA: Root mean square error of approximation
SRMR: Standardized root mean square residual
